# Metabolic Contributions of Wnt Signaling: More Than Controlling Flight

**DOI:** 10.3389/fcell.2021.709823

**Published:** 2021-09-09

**Authors:** Frederic Abou Azar, Gareth E. Lim

**Affiliations:** ^1^Department of Medicine, Université de Montréal, Montreal, QC, Canada; ^2^Cardiometabolic Axis, Centre de Recherche du Centre Hospitalier de l’Université de Montréal (CRCHUM), Montreal, QC, Canada

**Keywords:** Wnt signaling, β-catenin, TCF7L2, GSK-3β, metabolism, embryogenesis, organogenesis

## Abstract

The canonical Wnt signaling pathway is ubiquitous throughout the body and influences a diverse array of physiological processes. Following the initial discovery of the Wnt signaling pathway during wing development in *Drosophila melanogaster*, it is now widely appreciated that active Wnt signaling in mammals is necessary for the development and growth of various tissues involved in whole-body metabolism, such as brain, liver, pancreas, muscle, and adipose. Moreover, elegant gain- and loss-of-function studies have dissected the tissue-specific roles of various downstream effector molecules in the regulation of energy homeostasis. This review attempts to highlight and summarize the contributions of the Wnt signaling pathway and its downstream effectors on whole-body metabolism and their influence on the development of metabolic diseases, such as diabetes and obesity. A better understanding of the Wnt signaling pathway in these tissues may aid in guiding the development of future therapeutics to treat metabolic diseases.

## Introduction

During embryogenesis, the Wnt signaling pathway has active and critical roles in tissue development, including, but not limited to, axon guidance, body segmentation, limb development, and stem cell differentiation (Yang, 2003; Dunty et al., 2008; Geetha-Loganathan et al., 2008; Nusse, 2008; Stanganello et al., 2019). While its importance during embryogenesis cannot be understated, the influence of active Wnt signaling on the postnatal growth of tissues and their physiological functions are now gaining wide appreciation. Some of the lesser appreciated functions of the Wnt signaling pathway are its influence on systemic, organ, and tissue metabolism and energy homeostasis.

Whole-body metabolism is regulated by various tissues, including brain, liver, pancreas, muscle and adipose, and each contributes significantly to carbohydrate and/or lipid metabolism. The brain requires a constant uptake of oxygen and glucose, where astrocytes convert glucose to lactate and neurons use glucose and lactate in an oxidative manner (Deitmer et al., 2019). The liver is a critical metabolic tissue, as it is capable of glucose and glycogen metabolism, gluconeogenesis, fatty acid metabolism, and drug metabolism (Rui, 2014). The pancreas is responsible for secreting digestive enzymes and hormones, most notably insulin and glucagon, to control glucose homeostasis (Roder et al., 2016). Skeletal muscles require glucose and fatty acids to function, and can themselves be metabolized and converted to amino acids for use by other tissues. Insulin promotes glucose uptake in muscle where it can also be stored as glycogen (McPherron et al., 2013). Adipose tissue not only functions as energy reservoirs, but also secretes key hormones and metabolites to help control systemic energy balance (Choe et al., 2016). For example, it releases leptin to promote satiety (McDuffie et al., 2004). Thus, the goal of this review is aimed at highlighting the important contributions of the Wnt signaling pathway and its effectors within these metabolic tissues.

## Overview of the Wnt Signaling Pathway

The naming of Wnt is derived from the gene *wingless (wg)* in *Drosophila melanogaster*, where its mutation resulted in a wingless phenotype (Sharma and Chopra, 1976). Its vertebrate homolog *Int1* encodes for a retrovirus docking site and was first discovered in mice (Nusse and Varmus, 1982). Wnts are thought to primarily mediate their effects via autocrine and paracrine mechanisms, as the hydrophobic palmitate group near the N-terminus serves to tether the protein to the plasma membrane of its secreting cell (Kaemmerer and Gassler, 2016). However, recent findings have detected Wnt within exosomes, which suggests that Wnts could exert effects in distal sites in the body (Gross et al., 2012; Beckett et al., 2013).

**FIGURE 1:**
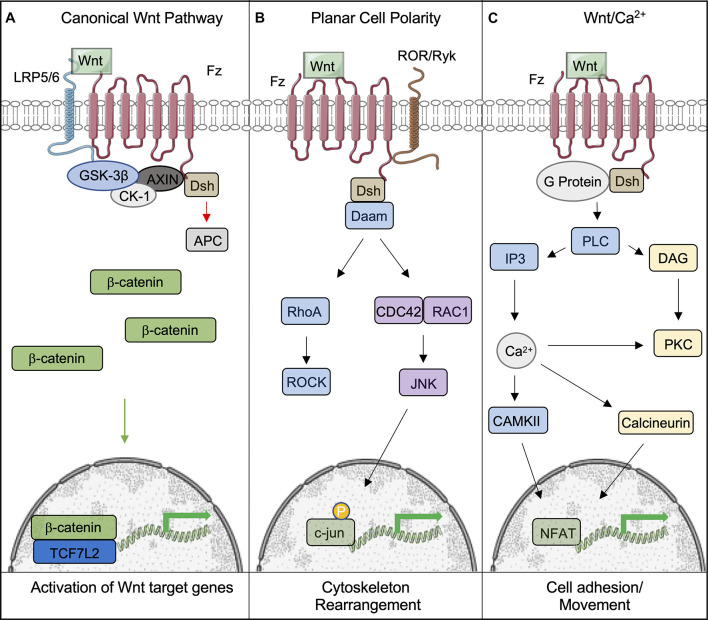
Overview of the canonical and non-canonical Wnt signaling pathways. **(A)** Following binding of Wnt to the Frizzled-LRP5/6 complex and the activation of the signaling pathway, the destruction complex (comprising of GSK-3β, CK-1, AXIN, and APC) is tethered to the plasma membrane, which permits β-catenin to accumulate and translocate to the nucleus, where it bind to TCF7L2 to activate downstream genes. **(B,C)** Wnts can also signal through non-canonical pathways to induce cell responses. **(B)** The Planar Cell Polarity pathway is activated upon Wnt binding to the Fz receptor. Dsh is subsequently activated, initiating the activites of Rho and Rac through Daam1. This leads to the eventual activation of ROCK and Jnk, which phosphorylates c-jun within the nucleus and allows it to initiate the transcription of target genes. **(C)** The Wnt/Ca^2+^ pathways is also activated by Fz; however, it is coupled to a G-protein, leading to intracellular Ca^2+^ release through PLC/IP3 activity. Ca^2+^ promotes the activity of CAMKII and Calcineurin to provoke the accumulation of the transcription factor NFAT in the nucleus. This figure was made with modified images from Servier Medical Art (Creative Commons Attribution 3.0 Unported License).

The initiation of the canonical Wnt signaling pathway, sometimes referred to as the Wnt/β-catenin pathway, occurs by the binding of Wnt proteins to complexes consisting of the cell-surface Frizzled (Fz) receptor and lipoprotein-related proteins (LRP5/6). This leads to a cascade of downstream events, that culminate in the dissociation of the β-catenin “destruction complex” comprised of Glycogen Synthase Kinase 3 (GSK-3), Axin, Casein-kinase 1 α (CK1-α), and Adenomatosis Polypsis Coli (APC) (Figure 1; Thompson and Williams, 2018). In the absence of Wnt signaling, GSK-3 binds β-catenin, the transcriptional co-activator integral to Wnt signaling, to promote its phosphorylation. The interaction of β-catenin with GSK-3 is initiated by CK1-α-mediated phosphorylation of Ser47, which allows GSK-3 to bind and subsequently phosphorylate additional serine and threonine residues (Thompson and Williams, 2018), leading to the eventual ubiquitination and degradation of β-catenin. Disheveled (DSH) is also recruited to the Fz-LRP5/6 complex and recruits the components of the destruction complex to the cell membrane (MacDonald and He, 2012). This aids in the stabilization and accumulation of β-catenin in the cytoplasm. Once β-catenin accumulates in the cytosol, it translocates to the nucleus where it binds the TCF family of transcription factors that influence downstream Wnt target genes and those that exert negative- or positive-feedback effects on the Wnt signaling pathway itself (Jho et al., 2002). The mechanism by which β-catenin enters and exits the nucleus is currently unknown, although some studies suggest the involvement of the scaffold protein 14-3-3ζ (Li et al., 2008).

Wnts can also bind to Fz and activate the non-canonical pathway, also known as the β-catenin-independent pathway. Non-canonical Wnts include Wnt1, Wnt2, Wnt5a, Wnt5b, and Wnt11 (Nie et al., 2020). This sub-grouping includes two distinct pathways called the Planar Cell Polarity (PCP) and Wnt/Ca^2+^ pathways (Komiya and Habas, 2008). PCP was discovered through loss of function studies of Fz and Dvl in *Drosophila*. The ensuing phenotype displayed randomized orientations of epithelial structures (Seifert and Mlodzik, 2007). This pathway has also been shown to be involved in cytoskeletal reorganization and coordination of cellular polarization (Komiya and Habas, 2008). It is activated by Wnt binding Dsh and Fz, which activates Rho through Daam1, activating the Rho kinase. Daam1 also activates Rac and JNK to initiate transcription via the actions of c-jun (Le Floch et al., 2005). The Wnt/Ca^2+^ pathway is activated by the same cytoplasmic receptors; however, it is coupled to a G-protein, leading to intracellular Ca^2+^ release by the actions of PLC and IP3 (Kohn and Moon, 2005). Collectively, the actions of CAMKII and Calcineurin lead to the accumulation of NFAT in the nucleus to initiate the transcription of target genes (Saneyoshi et al., 2002). This pathway is believed to affect early gastrulation cell movements and in some cases may inhibit the canonical Wnt pathway (Gilland et al., 1999; Wallingford et al., 2001).

Additional negative and positive modulators of the canonical pathway have been identified. For example, Frizzled-related protein family (SFRP) proteins and Wnt inhibitory factors, such as Dickkopf (DKK1), bind LRP5/6 directly to inhibit its association with the Fz receptor (Niehrs, 2006). On the other hand, antagonists like Cerberus bind Wnts extracellularly, inhibiting their ability to complex with the Fz/LRP5/6 receptor complex (Piccolo et al., 1999). Corepressors like Groucho/TLE, MTGR1 or COOP exert inhibitory effects by dissociating β-Catenin from TCFs (Cavallo et al., 1998; Moore et al., 2008; Song et al., 2010; Valenta et al., 2012). Nuclear APC has also been reported to sequester β-Catenin and prevent its interactions with TCFs (Hamada and Bienz, 2004). Positive modulators like serine-threonine phosphatase 2A (PP2A) increase β-Catenin’s activity by antagonizing the effects of GSK3 through de-phosphorylation of β-Catenin (Kimelman and Xu, 2006). This allows β-Catenin to translocate into the nucleus without being degraded. PP2A is also able to dephosphorylate Axin and prevent its association with β-Catenin (Willert et al., 1999). When taken together, multiple endogenous mechanisms exist that exert inhibitory effects on Wnt-β-catenin signaling.

## Roles of Wnt Signaling in the Development of Metabolic Tissues

**FIGURE 2:**
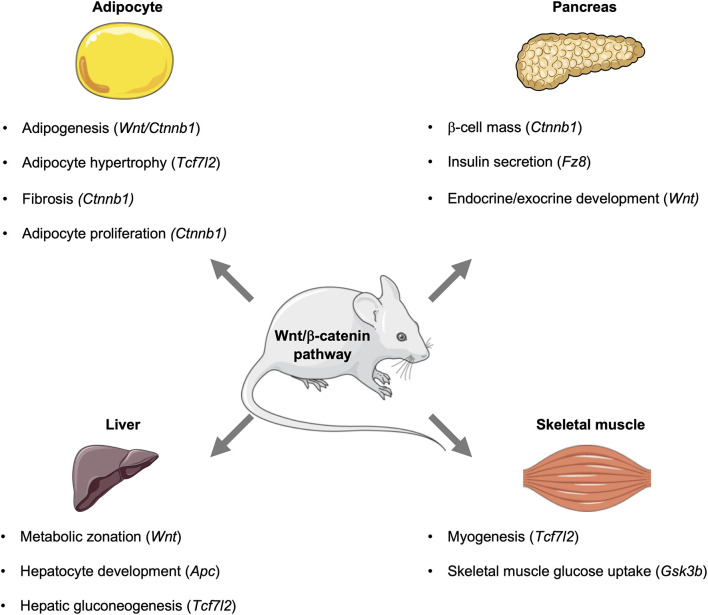
Influence of the Wnt signaling pathway and its downstream effectors in metabolic tissues. In mice, the Wnt signaling pathway influences metabolic events in key tissues, including the pancreas, adipose, liver, and skeletal muscle. Activation of this pathway can influence the development of tissues and organs, as well as signaling events that control glucose homeostasis and metabolism. This figure was made with modified images from Servier Medical Art (Creative Commons Attribution 3.0 Unported License).

Through the use of genetic approaches, the contributions of different cell types and tissues, such as adipose tissue, skeletal muscle, brain, pancreas, and liver, to overall metabolic homeostasis have been identified (Kitamura et al., 2003; von Maltzahn et al., 2012; Kubota et al., 2017), and Wnt signaling, which influences spatial patterning and organogenesis, is required for the development of these metabolic tissues (Tables 1–3 and Figure 2).

**TABLE 1:** Developmental phenotype resulting from over-expression or deletion of Wnt signaling effectors in *Drosophila*.

Effector	Model	Effect	Reference
Wingless	*wgIL114*	– Impaired protocerebrum development – Apoptosis of ventral ganglia cells	Richter et al., 1998
	HSwg/TM3, hb-LacZ	– Doubling of cerebral mass	Richter et al., 1998; Lee et al., 2014
	*wgIIID23*	– Absence of fat body in ventral section – Enhanced fat body in dorsolateral segment	Riechmann et al., 1998

### Wnts

Numerous mammalian Wnt proteins have been identified, with each isoform having distinct physiological functions in different tissues [for a detailed list, please refer to van Amerongen and Berns (2006)]. Deletion of one allele of a given Wnt does not necessarily lead to an overt phenotype, as demonstrated by the outcome of homo- and hetero-zygous *Wnt1* deletion on brain development and viability (Thomas and Capecchi, 1990; van Amerongen and Berns, 2006). The Wnt ortholog in *Drosophila*, *wg*, has been shown to be important for brain development (Table 1; Richter et al., 1998), as deletion of *wg* in fly embryos resulted in partial development of the protocerebrum. Loss of function of *wg* also led to enhanced apoptosis in the ventral ganglia, and gain-of-function experiments using a hsp70 promoter exhibited an increase in CNS development in flies, with a two-fold increase in cerebral mass (Richter et al., 1998).

Aside from roles in brain development, Wnt ligands also have essential roles in primitive streak formation and at various stages of embryogenesis (van Amerongen and Berns, 2006). For example, Wnt7a mediates the formation of ventral cell types during limb development in the central nervous system of mice, whereas Wnt4 is involved in the formation of the anterior-posterior axis during spinal cord development and is responsible for directing the migration of neurons and their axons (Parr et al., 1993; Hollis and Zou, 2012).

Orthologs of Wnt also exist in different species, where they exhibit evolutionarily conserved functions. For example, in *C. elegans*, the orthologous *Wnt* gene, *mom-2* is responsible for maintaining polarity of the embryo during development (Bischoff and Schnabel, 2006). In mammals, several Wnt proteins have also been found to have similar roles in maintaining cell polarity and the development of various metabolic organs, as seen by deletion of Wnt3 that results in the failure of Anterior-Posterior axis formation in the developing vertebrate embryo (Liu et al., 1999). Deletion of Wnt proteins are also associated with defects in organogenesis, as demonstrated by the failure of mesenchymal stem cells to differentiate into epithelial cells in kidneys of mice deficient in Wnt4. This results in the inability to form nephrons (Stark et al., 1994).

In the early stages of *Xenopus* embryo development (Table 2), inhibition of the Wnt signaling pathway appears to be essential, as injection of mRNA for *dkk-1*, the Wnt inhibitor, into blastomeres of *Xenopus* embryos led to anteriorized embryos displaying large heads, enlarged cement glands, and short trunks (Glinka et al., 1998). On the other hand, embryos injected with antibodies targeting *dkk-1* exhibited the opposite phenotype of microcephaly accompanied with a lack of cement glands. The embryonic axis remained unaffected, highlighting the importance of Wnt inhibition for *Xenopus* brain development. Toward the later stages of development, Wnt signaling is necessary for the downregulation of BMP-4 expression to induce the formation of the dorsal ectoderm (Baker et al., 1999). Antagonizing BMP-4 further allows for neural induction, a function found to be mediated by TCF7L2. For a more detailed anaylsis of the Wnt signaling pathway in the developing *Xenopus* brain, please see the review by Patapoutian and Reichardt (Patapoutian and Reichardt, 2000).

**TABLE 2:** Developmental phenotype resulting from over-expression or deletion of Wnt signaling effectors in *Xenopus.*

Effector	Model	Effect	Reference
Dickkopf	mRNA injection	– Anteriorized embryos – Large heads – Short trunks	Glinka et al., 1998
	Antibody targeting Dkk-1	– Microcephaly – Lack of cement glands	Glinka et al., 1998
Wnt8	Plasmid injection in D1 cells	– Inhibited development of pancreas, liver, and intesting	Schohl and Fagotto, 2002
	dnWnt8 in dorsal marginal zone of gastrula	– Induced MyoD expression	Hoppler et al., 1996
β-Catenin	mRNA injection	– Supressed foregut markers	Schohl and Fagotto, 2002
GSK3B	mRNA injection in lateral region of embryo	– Diminished expression of MyoD and Myf5	Shi et al., 2002

Wnt proteins are highly expressed in neurons in the posterior segment of the *Planarian* brain (Adell et al., 2009). They plays an inhibitory role on brain expansion, as evidenced by the increase in brain cells following RNAi mediated suppression of *wnt11-6* (Kobayashi et al., 2007; Hill and Petersen, 2015). Effectors of the Wnt signaling pathway can also affect organogenesis independent of β-catenin, as RNAi-mediated silencing of Dvl in *Planarians* compromised the development of the posterior ectopic brain (Almuedo-Castillo et al., 2011). Knockdown of β-catenin led to trunk loss, lack of tail identity, and loss of gut anterior-posterior polarity. However, brain development appeared to be phenotypically normal, indicating Dvl exerts its effects through a non- canonical Wnt pathway (Gurley et al., 2008).

During embryogenesis, various Wnt genes exhibit a biphasic pattern of expression. Some family members are upregulated before embryonic day-15 (e15) in mouse embryos, followed by their rapid downregulation by e16 (Heller et al., 2002). Wnt1 over-expression under the control of the *Pdx1* promoter disrupted spleen development and caused pancreas agenesis (Heller et al., 2002; Table 3). Similarly, over-expression of Wnt5a, the non-canonical Wnt, also altered pancreatic development, resulting in reduced pancreas size and the production of atypical endocrine structures (Heller et al., 2002), showing that both Wnt pathways can share common functions. Another example of the requirement of Wnt signaling in pancreatic development has been observed in mice expressing a dominant-negative Fz receptor (*Pdx1-Fz8CRD*) (Papadopoulou and Edlund, 2005). Transgenic mice expressing the dominant-negative FZ receptor in PDX1-positive cells displayed reduced pancreatic mass, but did not develop hyperglycemia or diabetes. β-cells of transgenic mice had a 50% increase in insulin content, suggesting a compensatory mechanism for the loss of pancreatic mass. The reduction in pancreatic mass of transgenic mice is partially attributed to decreased pancreatic cell proliferation (Papadopoulou and Edlund, 2005).

**TABLE 3:** Developmental phenotype resulting from the over-expression or deletion of Wnt signaling effectors in mice.

Effector	Model	Effect	Reference
Wnt1	OE (*Pdx1*-driven transgenic)	Agenesis of pancreas and spleen	Heller et al., 2002
Wnt5a	OE (*Pdx1*-driven transgenic)	Reduced size of pancreas, stomach, and duodenum	Heller et al., 2002
Frizzled receptor-8 (Fz8)	OE (*Pdx1*-driven expression of a dominant negative)	– Reduced pancreatic mass – Increased insulin secretion and content in β-cells – Decrease in pancreatic cell proliferation	Papadopoulou and Edlund, 2005
β-Catenin	*Pdx1*-Cre: *Ctnnb1*^flox/flox^	– Embryonic lethality – Acinar hypoplasia	Wells et al., 2007
	OE (*aP2*-Cre driven constitutively active)	Fibrosis in subdermal tissue	Zeve et al., 2012
	OE (*Pparg*-tTA; TRE-Cre driven constitutively active)	– Reduction of VAT – Elevated serum triglyceride – Fibrosis in SCAT – Depletion of adipocytes	Zeve et al., 2012
APC	*Ngn3*-Cre: *APC*^flox/flox^	Pancreas lacking β/α-cells	Sharon et al., 2019
	*Pdx1*-Cre: *APC*^flox/flox^	– Enlargment of pancreas – Acinar hyperplasia	Strom et al., 2007
TCF7L2	*Tcf7l2* ^GFPCre+neo/fl^	Mediates myogenic maturation	Mathew et al., 2011
	*Pax7-*Cre: *Tcf7l2*^flox/flox^	Reduction in hind limb and diaghpragm myogenesis	Mathew et al., 2011

*KO, knockout; OE, over-expression.*

In the liver, Wnt ligands have been found to influence liver growth and regeneration, as well as regulating cell proliferation and apoptosis (Monga, 2011). Various Wnt ligands, are expressed in the liver during development (Lade and Monga, 2011), and while it is not known if all Wnts have roles in liver development, Wnt2b has been implicated in liver organogenesis during the embryogenesis (Ober et al., 2006). The conservation of Wnt2b in liver development has also been observed in zebrafish embryos, as morpholinos against *wnt2bb* led to agenesis of hepatic tissue (Ober et al., 2006). The Wnt signaling pathway is active in *Xenopus* blastula as well; however, its activity decreases during gastrulation, suggesting it is not essential for early stage organogenesis (Schohl and Fagotto, 2002). Development of the pancreas, liver and intestine can be impaired by injection of Wnt8 plasmids into D1 cells of late stage *Xenopus* embryos. Moreover, micro-injection of stabilized forms of β-catenin mRNA into blastomeres were sufficient to supress foregut markers and recapitulated the phenotypes observed under Wnt8 administration. Administering DKK1 enhanced expression of *for1* and *pdx1*, markers of the liver and pancreas, respectively. Enlarged liver and pancreas buds were observed at stage 42 of embryogenesis. Similarly, overexpression GSK3-β enhanced expression of *for1* and *pdx1* (Schohl and Fagotto, 2002).

One critical function of the hepatic Wnt signaling pathway is its role in liver zonation, which is the distribution of proteins along a protocentral axis defined by periportal (PP) and perivenous (PV) hepatocytes (Sekine et al., 2006; Yang et al., 2014). PP hepatocytes are situated near the portal vein and are exposed to high concentrations of oxygen and nutrients when compared to PV hepatocytes. Thus, enzymes requiring a high demand of oxygen and nutrients, such as gluconeogenic enzymes, are predominantly expressed in in PP hepatocytes, as they are more aerobic than PV hepatocytes (Jungermann and Katz, 1989; Braeuning et al., 2006). Meanwhile, enzymes involved in glutamine synthesis are enriched in PV hepatocytes, which provide glutamine to PP hepatocytes for urea biosynthesis (Jungermann and Katz, 1989).

Claudin-2 is a tight junction protein important for the regulation of bile composition and flow (Ma, 2020), and deletion of Claudin-2 in mice results in decreased bilary flow and impaired generation of the osmotic water gradient in bile ducts (Matsumoto et al., 2014). While it is predominantly located in PV hepatocytes, its expression pattern can be altered by inhibiting Wnt exocytosis by deleting *Wls*, the gene encoding the Wnt receptor GPR177, in murine endothelial cells (Ma, 2020). This results in decreased Claudin-2 expression in adult PV hepatocytes (Ma, 2020). However, more recent studies suggest Wnt signaling may also play a role in liver zonation of newborn mice (Boj et al., 2012). Microarray analysis of hepatocytes isolated from whole-body TCF7L2 knockout mice revealed differential expression of liver zonation genes (Boj et al., 2012). In particular, *Glu1* (Glutamine synthase) and *Rnase4* (Ribonuclease 4) were found to be upregulated at the neonatal stage, with no differences during the embryonic phase (Boj et al., 2012). These studies highlight the importance of the Wnt signaling pathway in postnatal liver development and zonation, as well as demonstrating cellular crosstalk between hepatocytes and endothelial cells to regulate Wnt activity in adult hepatocytes.

Myogenesis is the development of muscle tissue, originating in precursor satellite cells expressing PAX3/7. Satellite cells undergo commitment and differentiation into myoblasts, followed by differentiation to myotubes. The fusion of myotubes leads to the formation of specific muscle fibers (Buckingham et al., 2003; Ganassi et al., 2018; Ultimo et al., 2018). Each step of myogenesis requires the input of multiple signaling pathways that control the expression of myogenic regulatory factors, such as Myogenin, MyoD and Myf5 (Zammit, 2017), and the Wnt signaling pathway influenced the expression of these myogenic factors (Ridgeway et al., 2000; Vertino et al., 2005). Whole-body deletion of Wnt10b in mice is associated with increased myogenic potential, as defined by higher levels of MyoD and Myogenin, and this is partially mediated by an upregulation of Wnt7b (Vertino et al., 2005).

*Xenopus* myogenesis requires enhanced Wnt signaling activity, as dominant-negative Wnt-8 supresses induction of MyoD in embryos. Gain of function experiments in the dorsal marginal zone of the gastrula showed ectopic expression of Wnt8 is sufficient to induce MyoD expression (Hoppler et al., 1996). GSK3-β mRNA injections in the lateral region of embryos diminished expression of MyoD and Myf5, independent of their expression in the dorsal mesoderm (Shi et al., 2002). These studies suggest Wnt has local, specific effects on myogenesis during embryogenesis.

Formation of neuromuscular junctions, which are responsible for the contraction of skeletal muscle, are also dependent on Wnt ligands. Acetylcholine receptors (AChRs) are highly enriched within neuromuscular junctions (Cisternas et al., 2019), and over-expression of Wnt3 in chick wing muscles increased AChR aggregates by up to 70% (Henriquez et al., 2008). In contrast, inhibition of the Wnt signaling pathway due to over-expression of Sfrp1 decreased the number of AChR aggregates and reduced the formation of neuromuscular junctions (Henriquez et al., 2008). The actions of Wnt3 were mediated by a non-canonical Wnt signaling mechanism that required the actions of the Rho GTPase, Rac (Henriquez et al., 2008). Collectively, these findings indicate Wnt ligands are required for skeletal muscle formation and function.

### β-Catenin

β-Catenin is a member of the Armadillo protein family and has been shown to have pleiotropic functions, including cell adhesion to gene transcription. One of the earliest, identified functions of β-catenin is its involvement in cell-cell adhesion via its association with E-cadherin to form adherens junctions (Valenta et al., 2012). β-catenin has also been reported to serve as a transcriptional co-activator of the Wnt signaling pathway (Sineva and Pospelov, 2014). In early embryonic development, β-catenin is involved in establishing the body axis and is critical for tissue and organ development, including but not limited to enamel, lung, kidney, and cartilage (Eberhart and Argani, 2001; Grigoryan et al., 2008; Prakash and Swaminathan, 2015). In the postnatal period, β-catenin contributes to cell renewal, as it has been shown to mediate the regeneration of hair follicles and retinal cells (Grigoryan et al., 2008; Steinhart and Angers, 2018).

β-catenin is characterized by the presence of 12 Armadillo repeats, which help to form a rigid center with flexibility at the C- and N- termini (Huber et al., 1997). Armadillo repeats generally share 30% homology, and this provides β-catenin with the ability to have a wide range of binding partners, including proteins associated with its destruction complex, inhibitors of the Wnt pathway, and transcription factors, such as BCL9, CBP, and Pontin-52 (Peifer et al., 1994; Xu and Kimelman, 2007). Depending on its role in the cell, β-Catenin can undergo conformational changes or dimerize. For instance, dimerization with α-catenin occurs in the context of cell-cell adhesion, but with gene transcription, a monomeric, backfolded conformation is required (Pokutta and Weis, 2000; Gottardi and Gumbiner, 2004). The C-terminal domain has been found to be integral for signaling activities, whereas its Helix-C motif is required for adhesion (Xing et al., 2008). In fact, most of β-catenin’s binding partners, such as 14-3-3ζ, require a Helix-C motif recognition sequence (Fang et al., 2007; Mosimann et al., 2009). Lastly, the motif recognized by GSK3-β, which marks β-catenin for degradation, is located on the N-terminal domain (Wu et al., 2003). Mutations in this region of the N-terminal domain leads to a constitutively active form β-catenin, which is found in multiple cancers (Buendia, 2002; Nejak-Bowen and Monga, 2011; Lade et al., 2012).

Proteins related to β-catenin include plakoglobin (γ-catenin), δ-catenin, and α-catenin, and like β-catenin, they associate with E-cadherin (Zhao et al., 2011). Among the related proteins, only plakoglobin exhibits a high degree of homology to β-Catenin, and it also contains 12 Armadillo repeats. Evolutionary analyses have revealed that plakoglobin is derived from a gene duplication of β-Catenin in vertebrates (Zhao et al., 2011), and despite the high degree of homology, plakoglobin, unlike β-catenin, is primarily localized to desmosomes (Lewis et al., 1997). Instead of armadillo repeats, α-catenin contains 3 vinculin domains, which has led to its classification as its own catenin subfamily (Zhao et al., 2011). Although δ-catenin has ten Armadillo repeats, it belongs to its own subfamily of catenins, named the p120 family. δ-catenin’s primary binding partner is the NF-kB transcription factor (Perez-Moreno et al., 2006).

Deletion of β-catenin in PDX1-positive cells in the pancreas revealed that β-catenin is necessary for acinar development, but not for the development of endocrine cells. Knockout mice exhibited early lethality and were smaller in size than wildtype mice, and they developed pancreatic hypoplasia (Dessimoz et al., 2005; Murtaugh et al., 2005; Wells et al., 2007). Histological analysis of pancreata from β-catenin-deficient, PDX1-positive cells resembled hepatocytes, suggesting that trans-differentiation had occurred (Wells et al., 2007). Conversely, transgenic mice over-expressing a degradation-resistant form of β-Catenin displayed a hypoplastic pancreas that lacked endocrine and exocrine tissue when compared to wild-type controls (Heiser et al., 2006). Immunohistological analysis of pancreata revealed that these mice displayed significant reductions in PDX1-positive pancreatic progenitor cells and increased expression of the hedgehog signaling pathway receptor, PTCH1, which is known to regulate postnatal pancreatic β-cell mass (Heiser et al., 2006; Nakayama et al., 2008). Thus, modulating intracellular levels of β-catenin in the pancreas during embryogenesis can having lasting effects on the formation of a functioning pancreas.

β-catenin’s role in myogenic differentiation is not well-defined, as different groups have reported different effects of over-expression or depletion on the initiation of myogenesis (Cossu et al., 1996; Tajbakhsh et al., 1998; Goichberg et al., 2001; Mermelstein et al., 2007; Brack et al., 2008). Retroviral over-expression of β-catenin in C2 and L8 cells were sufficient to inhibit myogenesis by reducing the expression of myogenin (Goichberg et al., 2001), and this has been proposed to be mediated by adherens junctions, as myogenin levels were rescued through the co-transfection of N-cadherin (Goichberg et al., 2001). This observation was recently supported by the finding that dimerization of β-catenin with α-catenin at adherens junctions is necessary for myogenesis (Cui et al., 2019). Moreover, RNA sequencing of control and β-catenin-null myocytes treated with Wnt3a revealed an upregulation of myogenic genes in control cells but not in knockout cells (Cui et al., 2019). It should be noted that expression of a β-catenin mutant unable to bind TCF7L2 did not impair myogenesis, suggesting that β-catenin exerts its myogenic effects via a TCF7L2-independent manner (Cui et al., 2019). In contrast, depletion of β-catenin in C2C12 cells inhibited myotube differentiation (Kim et al., 2008), and β-catenin depletion in 10T1/2 fibroblasts was also found to reduce the expression of MHC, a marker of muscle cell differentiation, indicating an impairment in myogenesis (Kim et al., 2008). Critical to β-catenin’s role in myogenesis was the finding that interactions of β-Catenin to MyoD via its C-terminal domain was necessary for the transcriptional activity of MyoD (Kim et al., 2008).

Human CD56^+^ muscle cell progenitors express low levels of nuclear β-catenin prior to the induction of myogenesis, followed by a marked increase post-differentiation (Agley et al., 2017). Pharmacological inhibition of GSK-3β with BIO, CHIR, or LiCl was sufficient to inhibit differentiation, demonstrating inhibitory effects of active β-catenin myogenesis (Agley et al., 2017). Over-expression of a dominant-negative TCF7L2 in CD56^+^ cells also reduced intracellular levels of β-catenin and impaired the onset of myogenesis, which suggests that TCF7L2 is required for β-catenin-mediated myogenesis (Agley et al., 2017).

### Adenomatosis Polypsis Coli (APC)

In addition to its roles as a tumor suppressor and a component of the β-catenin destruction complex, APC is also responsible for regulating chromosome formation, DNA replication, cell cycle progression, and apoptosis (Groden et al., 1995; Morin et al., 1996; Green et al., 2005). Nonsense mutations in the *APC* gene, which are generally associated with the generation of truncated forms of APC, contribute to the development of colon cancer (Powell et al., 1992; Laken et al., 1999; Fearnhead et al., 2001; Hankey et al., 2018).

Liver-specific deletion of APC produces an inverted expression of β-catenin along the proto-central axis and is associated with hepatomegaly and increased mortality in mice (Benhamouche et al., 2006). Knockout mice were also found to have increased circulating ammonia and elevated glutamine in the brain, which is suggestive of hepatic encephalopathy (Benhamouche et al., 2006).

In contrast to β-catenin, which is responsible for the development of the exocrine pancreas, APC appears to be important for both endocrine and exocrine cells (Sharon et al., 2019). Deletion of APC in cells expressing NEUROG3, an endocrine marker in the pancreas, impaired pancreatic endocrine cell development, as insulin- and glucagon-containing β-cells and α-cells, respectively, failed to develop (Sharon et al., 2019). In contrast, ablation of APC in exocrine cells led to an enlargement of the pancreas, characterized by acinar cell hyperplasia, and no changes in glucose homeostasis, or insulin and glucagon content were detected (Strom et al., 2007). Overall, APC is able to regulate the development of both exocrine and endocrine cells of the pancreas.

### TCF7L2

TCF7L2 (also known as TCF4) is an important component of the Wnt signaling pathway, as it binds to β-catenin to mediate the expression of critical Wnt-target genes. Genome-wide association studies have revealed a strong association with SNPs close to the TCF7L2 locus and the risk of developing of type 2 diabetes (Grant et al., 2006; Boj et al., 2012). These SNPs have been associated with impaired insulin secretion and increased hepatic glucose production under periods of fasting (Florez et al., 2006; Saxena et al., 2006; Lyssenko et al., 2007; Boj et al., 2012).

Systemic deletion of TCF7L2 (TCF7L2KO) in mice results in early lethality, as newborns were considerably hypoglycemic 3 h postpartum (Boj et al., 2012). Notably, embryonic development of the endocrine pancreas was not affected, but glycogen storage, triglyceride synthesis, fatty acid oxidation, and ketone body synthesis were diminished in livers of TCF7L2KO mice (Boj et al., 2012). Indeed, *Gys2* (Glycogen Synthase 2) mRNA levels were decreased, while mRNA levels of *Pck1, G6pc*, and *Fbp1* were increased, which may account for changes in glycogen synthesis and gluconeogenesis at birth (Boj et al., 2012).

Postnatal deletion of TCF7L2 in β-cells was not associated with any effects on body weight or glucose homeostasis in mice fed normal chow or high-fat diets (Boj et al., 2012). Histological examination of pancreata did not reveal any differences in β-cell mass or β-cell proliferation, suggesting that TCF7L2-dependent Wnt signaling was not required for pancreatic β-cell development (Boj et al., 2012).

Deletion of TCF7L2 in hepatocytes (LTCF7L2KO) resulted in mild fasting hypoglycemia, similar to what was observed with systemic TCF7L2KO mice (Boj et al., 2012). Microarray analysis revealed diminished expression of genes involved in glycolysis, fatty acid metabolism, and in the Wnt signaling pathway in livers of LTCF7L2KO mice (Boj et al., 2012). Under high-fat diet conditions, LTCF7L2KO mice displayed a more severe hypoglycemic phenotype, as hepatocytes from LTCF7L2KO mice demonstrated impaired gluconeogenesis following glucagon or IBMX treatment and reduced mRNA levels of gluconeogenic genes *G6pc* and *Aldh3a2* (Boj et al., 2012). Opposite to the LTCF7L2KO mice, adenoviral-mediated over-expression of TCF7L2 increased fasting glucose concentrations, in addition to enhanced hepatic glucose production following pyruvate injections (Boj et al., 2012). Genes associated with gluconeogenesis, such as *Pck1, G6pc*, and *Fbp1*, require functional TCF7L2, as hepatic over-expression of a dominant-negative TCF7L2 mutant under the control of the *Alb* promoter, resulted in their upregulation (Ip et al., 2015). Moreover, over-expression of the TCF7L2 mutant was associated with a progressive worsening in gluconeogenesis and glucose tolerance, despite no changes in insulin sensitivity (Ip et al., 2015).

In skeletal muscle, TCF7L2 expression is low when compred to other tissues (Osmark et al., 2009); however, TCF7L2 appears to be highly expressed in muscle connective tissue fibroblasts (Mathew et al., 2011). Fibroblast-specific deletion of TCF7L2 in mice was associated with reduced expression of *Myh7*, a marker of myogenic maturation, in slow-muscle fibers. However, its expression was upregulated in the skeletal muscle of adult mice, with the exception of the soleus muscle, suggesting that TCF7L2 exerts developmental effects on myogenic maturation. Deletion of TCF7L2 in fetal myogenic cells using a *Pax7-*Cre driver was associated with reduced *Myh7* and *Myh2* gene expression in hind limb muscles and the diaphragm, supporting the hypothesis that TCF7L2 is important for myogenesis (Mathew et al., 2011).

## Contributions of Wnt-β-Catenin Signaling Effectors to Metabolism

Since establishing the roles of Wnt signaling and its downstream effectors in the development of metabolic tissues and organs, the individual contributions of effectors to cellular and whole-body metabolism is now gaining wide appreciation. This includes their metabolic contributions in various metabolic organs and tissues, including liver, brain, pancreas, and adipose tissue (Tables 4, 5 and Figure 2).

**TABLE 4:** Metabolic phenotype resulting from the over-expression or deletion of Wnt signaling effectors in mice.

Effector	Model	Effect	Reference
β-Catenin	*Pdx1*-Cre: *Ctnnb1*^flox/flox^	– Fasting hyperglycemia – Reduced glucose clearance – Increased insulin resistance	Elghazi et al., 2012
	OE (*Pdx1*-driven expression of a constitutively active)	– Hypoplasic pancreas – Lacking endocrine and exocrine tissue	Heiser et al., 2006
	OE (Ad-Cre: *Ctnnb1*^flox/flox^)	– Reduced hepatic glucose production – Improved insulin tolerance – Improved glucose tolerance	Liu et al., 2011
	OE (adenovirus in hepatocytes)	– Stimulate hepatic gluconeogenesis	Liu et al., 2011
	OE (*RIP*-Cre driven)	– Expansion of β-cell mass – Decreased fasting glucose	Rulifson et al., 2007
	*Adipoq*-Cre: *Ctnnb1*^flox/flox^	– Reduced body weight – Reduced body size – Improved insulin sensitivity	Chen et al., 2019
	OE (*aP2*-Cre driven constitutively active)	Fibrosis in subdermal tissue	Zeve et al., 2012
	OE (*Pparg*-tTA; TRE-Cre driven constitutively active)	– Reduction of VAT – Elevated serum triglyceride – Fibrosis in SCAT – Depletion of adipocytes	Zeve et al., 2012
	*HAS*-MCM-Cre: *Ctnnb1*^flox/flox^	– Reduced insulin-stimulated glucose transport – Mild glucose intolerance – Mild insulin resistance	Masson et al., 2020
APC	*TTR*-Cre^Tam^: *APC*^flox/flox^	– Hepatomegaly – Increased mortality – Increase in circulating ammonia/glutamine levels in the brain	Benhamouche et al., 2006
TCF7L2	*Tcf7l2* ^–/–^	– Lethality – Hypoglycemia – Impaired hepatic function	Boj et al., 2012
	*RIP-*Cre-ER^T2^: *Tcf7l2*^flox/flox^	No effect	Boj et al., 2012
	OE (Ad-Cre: *Tcf7l2*^flox/flox^)	– Increase fasting glucose – Increase hepatic glucose production	Boj et al., 2012
	*SA-C*re*-ER^T2:^ Tcf7l2^f^*^lox/flox^	Mild fasting hypoglycemia	Boj et al., 2012
	*Adipoq-*Cre: *Tcf7l2*^flox/flox^	– Glucose intolerant – Hepatic insulin resistance	Chen et al., 2018
	*Adipoq-*Cre: *Tcf7l2*^flox/flox^	Weight gain	Geoghegan et al., 2019
	*Adipoq-*Cre: *Tcf7l2*^flox/flox^	Reduced insulin secretion	Nguyen-Tu et al., 2021
GSK3B	*Gsk3B* ^–/–^	Embryonic lethality	MacAulay and Woodgett, 2008
	*Gsk3B* ^–/–^	Embryonic lethality	Hoeflich et al., 2000
	OE (*RIP*-Cre driver, crossed with *Ir*^±^)	– Impaired glucose tolerance – Smaller β-cells	Liu et al., 2008
	*SA*-Cre: *Gsk3B*^flox/flox^	No effect	Patel et al., 2008
	*Mlc1f*-Cre: *Gsk3B*^flox/flox^	– Enhanced glucose sensitivity – Improved insulin sensitivity	Patel et al., 2008

*KO, knockout; OE, over-expression.*

**TABLE 5:** Metabolic phenotype resulting from over-expression or deletion of Wnt signaling effectors in *Drosophila.*

Effector	Model	Effect	Reference
Axin	FRT82B Axn127/TM6b	– Decreased adipogenesis – Reduction of diacylglycerol, free fatty acid and trigylcerides	Zhang et al., 2017
Wingless	Mef2-Gal4 Mhc-Gal4 Tin-Gal4	– Increased whole body triglyceride	Lee et al., 2014
Ck1-α	RNAi-mediated knockdown	Upregulation of haemolymph glucose	Ugrankar et al., 2015

### Wnt

Activation of Wnt signaling has been found to increase pancreatic β-cell proliferation and the expression of cell cycle genes via the actions of the transcription factor PITX2 (Table 4; Rulifson et al., 2007). β-cell-specific over-expression of a constituitively active form of β-catenin was found to stimulate β-cell expansion. Conversely, inhibition of Wnt signaling by over-expression of the Wnt signaling inhibitor, AXIN, led to β-cell hypoplasia and dysregulated islet architecture (Rulifson et al., 2007). Increasing or decreasing Wnt signaling was associated with enhanced or defective insulin secretion, respectively, along with changes in glucose tolerance (Rulifson et al., 2007).

Adipogenesis, or adipocyte differentiation, in white adipose tissue is a two-step process by which mesenchymal stem cells commit to preadipocytes, followed by differentiation into mature adipocytes. The differentiation of preadipocytes is governed by the transcription factors PPARγ and CEBP-α, which drive committed preadipocytes toward terminal differentiation (Cristancho and Lazar, 2011). In general, activation of the Wnt-β-catenin pathway in pre-adipocytes inhibits adipogenesis with the exception of the non-canonical Wnt5b-related signaling, which potentiates adipocyte differentiation of 3T3-L1 preadipocytes (Kanazawa et al., 2005; Cristancho and Lazar, 2011). Moreover, some Wnt proteins, such as Wnt6 and Wnt10, can also inhibit the maturation of thermogenic brown adipocytes (Tseng et al., 2005). Wnt proteins have also been reported to increase leptin production, as acute administration of Wnt3a to differentiated 3T3-L1 cells is sufficient to enhance leptin mRNA levels (Chen et al., 2015).

Wingless (*wg*) has also been implicated in obesity in fruit flies. Abdominal fat body mass was increased following knockdown of *wg* using a Mef2-Gal4 driver (Table 5; Lee et al., 2014). Mhc-Gal4 and Tin-Gal4 drivers were also used to supress *wg* in the muscle and heart, respectively, and in both models, whole body triglyceride content was increased (Lee et al., 2014). Overexpression of *wg* in muscle exhibited the opposite phenotype, with a decrease in total fat accumulation, indicating Wingless can function as a repressor of adipogenesis (Riechmann et al., 1998). Indeed, deletion of *wg* in embryos showed a complete absence of fat in the ventral section. Conversely, the dorsolateral segment displayed high expression of adipocyte progenitors. Thus, *wg* appears to be essential for ventral adipogenesis and is required to supress dorsolateral mesodermal fat development. Gonadal agenesis was also reported in the mutant embryos, suggesting all dorsolateral cells were converted to adipocyte progenitors (Riechmann et al., 1998).

Non-canonical Wnt pathways have also been implicated in metabolic diseases. Wnt5A was found to be upregulated in visceral adipose tissue (VAT) of obese individuals compared to subcutaneous adipose tissue (SCAT) (Fuster et al., 2015), and Wnt5a inhibition in mice fed a high-fat diet prevented the development of metabolic dysfunction, with a reduction in insulin and glucose levels (Fuster et al., 2015). However, no changes in body weight or body fat percentage were detected, suggesting no effects on adipogenesis. Interestingly, proinflammatory cytokines such as TNF-α and CCL2/MCP-1 were downregulated, indicating a potential reduction in inflammation. sFRP5, which sequesters and antagonizes Wnt5a was also found to produce anti-inflammatory effects in mice (Ouchi et al., 2010). Deletion of sFRP5 in leptin-deficient *Ob*/*Ob* mice on a high-fat diet elevated glucose and triglyceride levels. Additionally, increases in F4/80 cells were detected in epididymal white adipose tissue, indicating increased inflammation (Ouchi et al., 2010).

LRP6 is essential for Wnt binding to Fz in the canonical pathway, but it can also inhibit the non-canonical Wnt signaling pathway (Bryja et al., 2009). LRP6 mutant mice, with nucleotide substitutions at 10443 and 10445, were found to have an enhanced levels of cytokines, MPO, IL-6, and CD68, in the liver (Wang et al., 2015). Histological analysis revealed mutant mouse livers to have a greater accumulation of lipid droplets, indicative of hepatic steatosis. Modest reductions in β-catenin were detected in the liver, with increased expression of RhoA and ROCK2 mRNA, suggesting enhanced activity of the non-canonical pathway. Fibrosis was also noted in the liver of mutant mice following high-fat diet feeding. Notably, recombinant Wnt3a administration was able to rescue the mutant phenotype and reduced inflammation (Wang et al., 2015). These findings demonstrate contrasting and opposing functions of the canonical and non-canonical Wnt pathways within the same tissue.

### β-Catenin

Gain- and loss-of-function approaches have revealed that β-catenin can influence hepatic metabolism (Liu et al., 2011). Adenovrial-mediated depletion of β-catenin in hepatocytes attenuated hepatic gluconeogenesis, resulting in hypoglycemia. Conversely, over-expression of β-catenin had opposite effects on hepatic glucose production. Levels of mRNA corresponding to the rate-limiting gluconeogenic enzymes Phosphoenolpyruvate carboxykinase (*Pck)* and Glucose-6-phosphatase (*G6p*) *in vivo*, as well as in Hepa1-6 hepatoma cells, were decreased or increased following depletion or over-expression of β-catenin, respectively (Liu et al., 2011). Under high-fat diet feeding conditions, depletion of β-catenin in hepatocytes was associated with improved glucose tolerance and reduced hepatic gluconeogenesis (Liu et al., 2011).

In pancreatic endocrine cells, β-catenin is essential for β-cell function and survival. Targeted deletion of β-catenin in murine β-cells during embryogenesis resulted in severe hypoglycemia and premature death. Moreover, β-cells from knockout mice displayed impaired insulin release (Dabernat et al., 2009). In various *in vitro* β-cell models, acute depletion of β-catenin has also been shown to impair glucose-stimulated insulin secretion (GSIS) due to negative effects on re-modeling of the actin cytoskeleton (Sorrenson et al., 2016). Deletion of β-catenin in the exocrine pancreas resulted in mice that were smaller in size and weight when compared to controls, in addition to impaired development of exocrine tissues (Elghazi et al., 2012). Deletion of β-catenin in exocrine tissue was also associated with fasting hyperglycemia, in addition to lower rates of glucose clearance and insulin sensitivity (Elghazi et al., 2012). Depletion of β-catenin also led to impaired pancreatic function and growth, as knockout mice displayed reduced β-cell proliferation and β-cell mass (Wells et al., 2007; Elghazi et al., 2012).

Recently, β-catenin levels have been found to be elevated in subcutaneous white adipose tissue from individuals with obesity (Chen et al., 2019). Similarly in mice, over-feeding was sufficient to increase β-catenin expression in murine adipose tissue, suggesting pro-adipogenic or -obesogenic roles in adipose tissues (Chen et al., 2019). Indeed deletion of β-catenin in adipocytes was found to be protective against high-fat diet-induced weight gain and improved insulin sensitivity. The attenuation of fat mass expansion was associated with significant reductions in proliferating PDGFRA-positive pre-adipocytes (Chen et al., 2019).

Over-expression of β-catenin in mature adipocytes under the control of the aP2 promoter was associated with fibrosis in subdermal tissues, with no other significant changes under normal chow diet conditions (Zeve et al., 2012). In contrast, β-catenin over-expression in adipocyte progenitor cells led to significant reductions in visceral white adipose tissue and circulating levels of leptin and adiponectin. Interestingly, over-expression of β-catenin in adipocyte progenitor cells was not associated with glucose intolerance or insulin resistance, as would be expected from a lipodystrophic phenotype (Zeve et al., 2012).

Inhibitors of the Wnt/β-catenin pathway have been shown to promote adipogenesis. For example, the β-catenin antagonist Chibby, which removes unphosphorylated β-catenin from the nucleus (Killoran et al., 2015), is necessary for adipogenesis. Depletion or ectopic over-expression of Chibby can inhibit or promote spontaneous adipogenesis, respectively (Li et al., 2007). Similarly, the Wnt antagonist, Dickkopf1 (DKK1), has similar effects in promoting adipogenesis. DKK1 inhibits the Wnt pathway by binding the receptors LRP5/6, and *in vitro* assays using human pre-adipocytes have shown that DKK1 expression increases during the early stages of adipogenesis (Christodoulides et al., 2006). In conditions associated with sub-optimial differentiation of 3T3-L1 adipocytes, ectopic expression of human DKK1 could restore differentiation to the same extent as complete adipogenic conditions due to inhibition of Wnt signaling (Christodoulides et al., 2006).

Skeletal muscle is responsible for 70–90% of glucose uptake from the circulation, and this process is impaired in individuals with type 2 diabetes (Evans et al., 2019). Although the Wnt signaling pathway is known to be involved in myogenesis, it has also been found to participate in skeletal muscle glucose metabolism. Deletion of β-catenin in skeletal muscle was found to diminish insulin-stimulated glucose transport in mice, and this was also associated with mild glucose intolerance and insulin resistance (Masson et al., 2020). *In vitro* studies with isolated muscle and myocytes demonstrate that knockdown of β-catenin can also reduce GLUT4 protein abundance upon insulin stimulation, demonstrating impaired insulin action (Masson et al., 2020).

### Axin

Removal of exon 11 of Axin (*Axn)* in *Drosophila* and its replacement with a repetitive heterochromatin sequence resulted in a hyperactive Wnt signaling response, as Armadillo (B -catenin’s *Drosophila* ortholog) expression increased (Zhang et al., 2017). Homozygous *Axn* mutants displayed decreased accumulation of abdominal adipocytes at mid to late larval stages, along with lower levels of diacylglycerol, free fatty acid, and triglycerides. Furthermore, RNAi-mediated knock down of Armadillo successfully rescued the phenotype (Zhang et al., 2017).

The axin ortholog in *C.elegans*, PRY-1, was recently shown to be involved in lipid metabolism (Ranawade et al., 2018). Worms with a nonsense *pry-1* mutation had enhanced Wnt activity, demonstrating that β-catenin activity on lipid metabolism is conserved across species. Mutants exhibited a reduction in lipid content, egg laying and survival following starvation. Genetic studies revealed a downregulation in *vits* and *fats*, the genes responsible for yolk lipoproteins and fatty acid desaturases (Ranawade et al., 2018).

### CK1-α

Ck1-α emerged as a candidate gene with high association to hyperglycemia in *Drosophila* (Ugrankar et al., 2015). RNAi-mediated knockdowns in adipose tissue or muscle of third instar larvae led to an increase in haemolymph glucose levels. Most notably, *CSNK1a1* knockouts in murine pre-adipocytes recapitulated the hyperglycemic phenotype, indicating a species-conserved function of CK1-α (Ugrankar et al., 2015).

### GSK3

GSK-3 is an important regulator of glycogen synthesis, as it is responsible for inhibiting glycogen synthase to attenuate glycogenolysis and glucose production (MacAulay and Woodgett, 2008). GSK-3 has two paralogs, GSK-3α and GSK-3β, and while systemic knockouts of GSK-3β are embryonically lethal due to enhanced TNF-α-associated hepatocyte apoptosis, systemic GSK-3α knockouts were found to be viable (Hoeflich et al., 2000; MacAulay and Woodgett, 2008). In addition, improved glucose tolerance, as well as higher insulin sensitivity, were observed, which may be attributed to increased mRNA levels of Glycogen Synthase and Insulin Receptor Substrate-1 (MacAulay and Woodgett, 2008).

Deletion of the insulin receptor (IR) in mice is known to cause perinatal lethality due to diabetic ketoacidosis, and mice heterozygous for *Ir* display mild insulin resistance and compensatory increases in β-cell mass. Partial deletion of the insulin receptor is also associated with significant hyperglycemia and hyperinsulinemia, despite exhibiting no changes in insulin signaling (Bruning et al., 1997). GSK3β is involved in the insulin signaling pathway, as it inactivates glycogen synthase by phosphorylating serine residues on its C-terminal domain. GSK3β can also inhibit the insulin pathway by phosphorylating the insulin receptor substrate-1 (IRS-1) directly (Liberman and Eldar-Finkelman, 2005). Mice heterozygous for *Gsk3b* (*Gsk-3*β^±^) exhibited decreased fed and fasting insulin levels, and when bred with mice heterozygous for *Ir*, haploinsufficiency of GSK3-β was able to inprove the metabolic phenotype of heterozygous *Ir* mice (Tanabe et al., 2008). Previous studies have shown that whole body-IRS2 knockout mice resemble insulin receptor knockout mice, as they displayed insulin resistance, hyperglycemia, reduced β-cell mass, and decreased body weight (Uchida et al., 2005). When compared to systemic IRS2 knockout mice, GSK-3β haploinsufficient mice on an *Irs2*-null background did not exhibit loss of β-cell mass due to increased β-cell proliferation and reduced rates of β-cell apoptosis (Uchida et al., 2005). Expansion of β-cell mass was regulated by reductions in the Cyclin-dependent kinase inhibitor, p27^Kip1^, a rate limiting factor in cell proliferation (Uchida et al., 2005; Rachdi et al., 2006).

Deletion of GSK-3β in β-cells is sufficient to partially rescue the diabetic phenotype of IRS2 knockout mice, as mice were normoglycemic despite being hyperinsulinemic (Tanabe et al., 2008). This was attributed to the deletion of GSK-3β resulting in reducing the incidence of apoptosis and promoting β-cell proliferation (Tanabe et al., 2008). Over-expression of a constitutively active GSK3-β (RIP-GSK3βCA) mutant in β-cells led to impaired glucose tolerance, which was only observed in male mice. Histological analysis of pancreata revealed that transgenic mice had decreased β-cell area, as well as impaired β-cell proliferation. Moreover, over-expression of a constituitively active GSK3-β mutant led to defects in insulin secretion and resulted in impaired glucose tolerance (Liu et al., 2008). In conjunction, these studies demonstrate the importance of GSK-3β in regulating β-cell mass and proliferation.

In liver-specific GSK-3β knockout mice, no significant metabolic changes were observed, thereby suggesting that GSK-3β in the liver is dispensable for whole-body metabolism (Patel et al., 2008). Skeletal muscle-specific knockout of GSK-3β mediated by *Mlc1f*-promoter driven Cre-mediated recombination results in mice with enhanced glucose tolerance. Morover, these mice had increased glycogen synthase, resulting in improved insulin signaling and actions, and this highlights GSK-3β’s importance in regulating insulin action in a tissue specific manner (Patel et al., 2008).

In the context of obesity, GSK-3β inhibition has been found to have beneficial metabolic effects. Administration of the GSK3 inhibitors SB216763 or CHIR99021 to high-fat diet-induced obese mice improved insulin sensitivity without any changes in fat mass or body weight (Wang et al., 2018). Furthermore, GSK-3 inhibition was found to influence macrophage polarization such that increased numbers of anti-inflammatory M2 macrophages could be detected (Wang et al., 2018). Additionally, *in vitro* studies performed on human adipose- derived stem cells showed that GSK3 inhibitors, such as LiCl and BIO, have the ability to inhibit cell proliferation, and BIO-treated human adipose-derived stem cells were unable to differentiate to adipocytes in the presence of adipogenic stimuli (Zaragosi et al., 2008).

### TCF7L2

Glucagon-like peptide-1 (GLP-1), which is derived from intestinal endocrine L cells, potentiates glucose-stimulated insulin secretion through actions on the GLP-1R on the surface of β-cells (Lim and Brubaker, 2006). In L cells, TCF7L2 participates in GLP-1 synthesis by regulating the transcription of *Gcg*, thereby representing an alternative mechanism whereby the Wnt signaling pathway can influence insulin secretion from β-cells (Shao et al., 2013). Indeed, targeted transgenic expression of a dominant-negative TCF7L2 mutant in neurons and enteroendocrine cells led to significant reductions in *Gcg-*positive neurons and GLP-1-positive cells, respectively (Shao et al., 2013, 2015). Although these mutant mice were found to be glucose intolerant and insulin resistant, compensatory increases in β-cell mass were detected (Shao et al., 2013). In adult mice, GLP-1 was found to induce the phosphorylation of β-catenin by cAMP/PKA or insulin/PAK-1 signaling pathways to facilitate β-cell proliferation (Xiong et al., 2012; Shao et al., 2013).

To examine TCF7L2’s effects on type 2 diabetes in zebrafish, heterozygous knockouts were generated by introducing a mutation in intron 1, which produced a truncated protein (Facchinello et al., 2017). Adult fish displayed postprandial hyperglycemia. Reduction of the overall size of the fish was observed, as well as reductions in the sizes of the endocrine and exocrine pancreas. Interestingly, the mutant exocrine pancreas was also found to have an accumulation of adipose tissue, along with a decrease in number of β-cells, suggesting a mechanism by which deficiency in TCF7L2 may lead to a diabetic phenotype protein (Facchinello et al., 2017).

**FIGURE 3:**
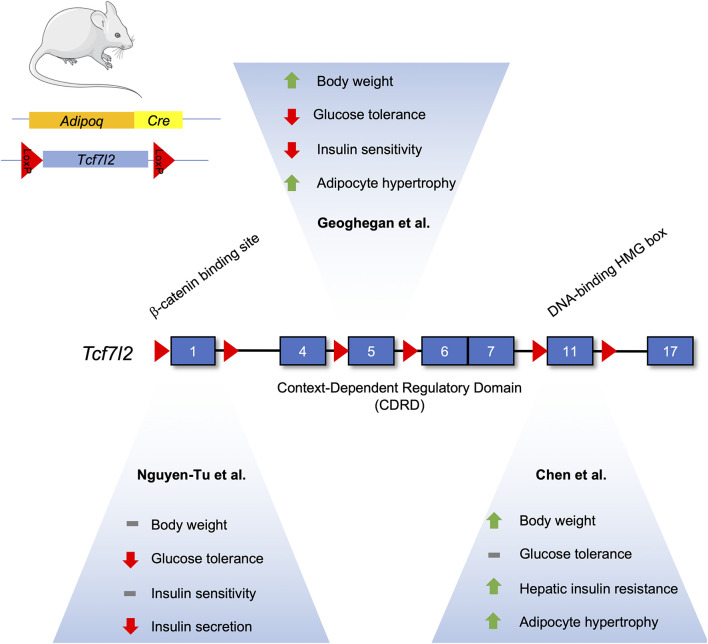
Differences in the metabolic phenotype of adipocyte-specific TCF7L2 knockout mouse models. The *Tcf7l2* gene contains 17 exons, as denoted by numerated blue rectangles. Recombination of floxed alleles for Exon 1 (Nguyen-Tu et al., 2021), Exon 5 (Geoghegan et al., 2019), and Exon 11 (Chen et al) by Cre recombinase under the control of the *Adipoq* promoter results in different metabolic phenotypes. This figure was made with modified images from Servier Medical Art (Creative Commons Attribution 3.0 Unported License).

TCF7L2 has been demonstrated to have important roles in adipogenesis, but it is not without controversy (Figure 3). One study suggests its expression increases during adipogenesis, which may seem contradictory as activated Wnt signaling is known to inhibit adipocyte differentiation (Cristancho and Lazar, 2011; Chen et al., 2018). Indeed, knockdown of TCF7L2 via short-hairpin RNA in 3T3-L1 cells inhibited adipogenesis and downregulated *Slc2a4* (GLUT4) mRNA. Adipocyte-specific TCF7L2 knockout mice were glucose intolerant and showed signs of hepatic insulin resistance that was associated with increased gluconeogenesis. Under high-fat diet conditions, knockout mice gained more weight and showed increased hypertrophy of inguinal white adipose tissue, and gene expression analysis of inguinal white adipose tissue displayed an increase in the Wnt target gene, *Axin2*, suggesting that the reduction of TCF7L2 activated the Wnt signaling pathway to initiate adipocyte hypertrophy.

In contrast, Geoghegan et al. (2019) have reported that TCF7L2 expression is reduced during adipogenesis in both *in vitro* and *in vivo* models. Mice fed a high fat diet exhibited an increased abundance of TCF7L2 and adiponectin expression in epididymal white adipose tissue, and this increase in TCF7L2 abundance could be recapitulated in adipose tissue from *ob/ob* and *db/db* mice (Geoghegan et al., 2019). Adipocyte-specific deletion of TCF7L2 led to weight gain when compared to control mice fed a normal chow diet. On a high-fat diet, knockout mice displayed significantly higher weight gain, in addition to impaired glucose tolerance and insulin resistance. Histological analysis revealed hypertrophy in inguinal WAT depots and lipolytic genes, such as *Tgh* and *Tgh2*, were downregulated. Adipocyte-specific knockout of TCF7L2 was associated with increased lipid storage, due in part to changes in *Tgh1* (triacylglycerol hydrolase) and *Tgh2* gene expression, and adipocyte hypertrophy (Geoghegan et al., 2019).

Further studies done by Nguyen-Tu et al. (2021) on TCF7L2 knockout mice using an *Adipoq*-Cre driver recapitulated the hyperglycemic phenotype seen by Chen et al. (2018). However, this phenotype was only seen in 16-week old males, and they were not insulin resistant. Insulin secretion was abrogated in isolated islet *ex vivo*, possibly due to the decreased islet expression of *Glut2* mRNA. No differences in insulin secretion were seen *in vivo* under normal chow-fed conditions, possibly due to compensatory increases in circulating levels of NEFAs that can potentiate glucose-stimulated insulin secretion. Furthermore, under high-fat diet, no weight changes were seen but TCFL2 knockout diminished insulin secretion (Nguyen-Tu et al., 2021).

All three studies examining TCF7L2’s effects on adipocyte function and adipogenesis were performed on male mice using different Adipocyte-specific Cre driver mice. However, the exons targeted for recombination were also different for all three studies. Chen et al., floxed exon 11, known to encode the DNA binding HMG box (Hansson et al., 2010). On the other hand, Geoghegan et al. (2019) used mice with lox-p sites flanking exon 5, while Nguyen-Tu et al. (2021) used exon 1 floxed mice. Both found TCF7L2 to have an inhibitory role on adipogenesis. Taken together, the common and differing phenotypes associated with TCF7L2 deletion demonstrate the complexity of the biological actions of the Wnt signalng pathway in adipocytes.

## Conclusion and Perspectives

Although the Wnt signaling pathway is conserved across species and is necessary for embryogenesis and development, it is now clear that it also has essential metabolic roles in mammals. Moreover, aberrant expression or activity of various downstream effectors in the Wnt signaling pathway is associated with the development of chronic diseases, including diabetes and obesity. Given the pathway’s wide ranging effects and multiple downstream targets, more research is required to further our understanding of its involvement across different metabolic diseases.

## Author Contributions

FAA wrote the manuscript. GEL edited the manuscript and the guarantor of this work. Both authors contributed to the article and approved the submitted version.

## Conflict of Interest

The authors declare that the research was conducted in the absence of any commercial or financial relationships that could be construed as a potential conflict of interest.

## Publisher’s Note

All claims expressed in this article are solely those of the authors and do not necessarily represent those of their affiliated organizations, or those of the publisher, the editors and the reviewers. Any product that may be evaluated in this article, or claim that may be made by its manufacturer, is not guaranteed or endorsed by the publisher.

## Abbreviations

APC: adenomatous polyposis coli
GSK: glycogen synthase kinase
TCF/LEF: transcription like factor lymphocyte enhancer factor
LRP: lipoprotein receptor-related protein
CK: casein kinase
Mtgr1: myeloid translocation gene related-1
PP: periportal hepatocytes
PV: perivenous hepatocytes
MyoD: myoblast determination protein
Myf: myogenic factor
NMJ: neuromuscular junction
AChR: acetylcholine receptor
BCL9: B-cell CLL/lymphoma protein 9
CBP: CREB-binding protein
NF-kB: nuclear factor kB
PTCH1: protein patched homolog 1
MHC: myosin heavy chain
LTCF7L2KO: liver specific TCF7L2 knockout
IBMX: isobutylmethylxanthine
PITX2: paired like homeodomain 2
MIN6: mouse INsulinoma 6 cells
CDK4: cyclin dependent kinase 4
PPAR: peroxisome proliferater activated receptor
CEBP: CCAT enhancer binding proteins
Brdu: bromodeoxyuridine
PDGFRa: platelet derived growth factor
DDR2: discoidin domain receptor
FSP-1: fibroblast specific protein 1
DKK1: Dickopf
TNF: tumor necrosis factor
IRS: Insulin receptor substrate
LiCl: lithium chloride
BIO: 6-bromoindirubin
GLP: glucagon like peptide
PKA: protein kinase A
cAMP: cyclic adenosine monophosphate
WAT: white adipose tissue
NEFA: non-esterified fatty acid
FABP: Fatty acid binding protein
HMG: high mobility group
DIO: diet induced obesity.
